# The Physician’s Bedside Record

**Published:** 1888-09

**Authors:** 


**The Physician's Bedside Record.** The Plympton Manufacturing Co., Hartford, Conn.

This convenient method of keeping the daily record of a case at the bedside commends itself to every careful, thorough, busy physician, and its cost, being a trifle, places it within the reach of all.

